# Timing of glaucoma treatment in patients with MICOF: A retrospective clinical study

**DOI:** 10.3389/fmed.2022.986176

**Published:** 2022-09-29

**Authors:** Zhao Li, Qun Wang, Shi-Feng Zhang, Yi-Fei Huang, Li-Qiang Wang

**Affiliations:** ^1^Department of Ophthalmology, The Chinese People's Liberation Army of China (PLA) General Hospital, Beijing, China; ^2^Department of Medical School, Nankai University, Tianjin, China

**Keywords:** MICOF, KPro, glaucoma, treatment, retrospective clinical study

## Abstract

**Purpose:**

To summarize and discuss the treatment and timing of glaucoma in patients with MICOF keratoprosthesis implantation to guide follow-up clinical treatment.

**Methods:**

The data of 39 eyes (39 patients) with the Moscow Eye Microsurgery Complex in Russia (MICOF) keratoprosthesis implantation in our hospital from 1 January 2002 to 31 December 2017 were collected, including patients with preexisting glaucoma and those who developed glaucoma *de novo* after MICOF. The sex, age, preoperative diagnosis, glaucoma surgery, keratoplasty, times of keratoplasty, best corrected visual acuity (BCVA) and final follow-up corrected visual acuity, visual field (VF) defect, and cup-to-disk ratio (CDR) were statistically analyzed.

**Results:**

Among 16 eyes with preexisting glaucoma, eight eyes underwent glaucoma surgery before MICOF, 4 eyes underwent glaucoma surgery combined with MICOF, and four eyes were managed medically. Among 23 eyes with *de novo* glaucoma, seven eyes were treated with surgery and 16 eyes were treated with medication only. A total of 9 (56.3%) eyes had corneal transplants with preexisting glaucoma, which was a higher percentage than that in the patients with *de novo* glaucoma (*n* = 5, 21.7%, *P* = 0.043). In both the preexisting glaucoma group and the *de novo* glaucoma group, the most common causes were alkali burns (56.3% of preexisting glaucoma and 43.5% of *de novo* glaucoma). There was no significant difference between the operation and initial visual acuity, postoperative visual acuity, BCVA, CDR, or VF defect. In the *de novo* glaucoma group, the final follow-up visual acuity of the glaucoma surgery group (1.56 ± 1.07) was worse than that of the mediation group (0.44 ± 0.53) (*P* < 0.017). Among the complications, the incidence of cornea melting in the patients treated with medications only (*n*=10) was significantly higher than that in the patients treated with glaucoma surgery (*n* = 0, *P* = 0.007), but there was no significant difference in the other complications.

**Conclusion:**

Among patients with MICOF, those patients who have undergone keratoplasty are more likely to develop glaucoma before surgery and glaucoma needs to be prevented. Surgical treatment can be selected according to the ocular surface condition in the patients with *de novo* glaucoma to reduce the occurrence of complications.

## Introduction

In some patients with severe corneal pathologies, such as chemical burns, autoimmune disease, Steven–Johnsons syndrome, and severe dry eye, amniotic membrane transplantation or corneal transplantation was mostly used in the past, but the curative effect was not ideal because of poor ocular surface conditions or inflammatory reactions caused by the sutures (1). Although it has been reported that the use of sutureless amniotic membrane transplantation improves the efficacy, large studies are still needed (2).

At present, keratoprostheses are developing rapidly, which provide more opportunities and choices for the abovementioned patients with severe disease to have their eyesight restored. There are three common types of keratoprosthesis: the Boston keratoprosthesis (Boston KPro), osteo-odonto keratoprosthesis (OOKP), and MICOF keratoprosthesis (developed by the Moscow Eye Microsurgery Complex in Russia thus, called MICOF) (3, 4). OOKP uses mucous membranes and alveolar bone to fix the optical cylinder (5), and the scope of operation is relatively large. The Boston KPro surgery is less invasive, but requires a donor cornea for support. However, MICOF does not require a donor cornea, and the surgical invasiveness is mild (6). MICOF may have greater application prospects. The gratifying thing is that MICOF has been clinically tested in our hospital and has achieved good results.

However, patients with all the types of keratoprostheses are at risk of glaucoma affecting vision. Preoperative assessment of glaucoma is affected by the transparency of the cornea, and the state of the ocular surface after keratoprosthesis implantation makes intraocular pressure (IOP) monitoring extremely difficult. Glaucoma is a blinding disease with a high incidence, so it is important to monitor the patients' relevant examination data to assess glaucoma progression and determine the appropriate glaucoma timing (7, 8).

Currently, the most research on the treatment of keratoprosthesis glaucoma is with the use of Boston KPro. Because the structures of keratoprosthesis are different, the treatment of MICOF glaucoma cannot be simply applied, and there are few studies on MICOF glaucoma. Our study analyzes the impact of preoperative diagnosis, and glaucoma surgery on the prognosis of glaucoma by evaluating the clinical data related to preexisting and *de novo* glaucoma in patients with MICOF, to draw experience for clinical treatment.

## Materials and methods

### Patients

Thirty-nine eyes of 39 patients were included in this retrospective study. The data of inpatients with MICOF in the Chinese PLA General Hospital from 1 January 2002 to 31 December 2017 were collected. All the patients underwent MICOF implantation, a two-stage surgery, which includes inserting a titanium frame and screwing a polymethyl methacrylate (PMMA) optical cylinder. All the surgeries were performed by two experienced surgeons. When there were no complications, the patients had the same medication regimen after the operation. All the patients had glaucoma. The studies involving human participants were reviewed and approved by. The study adhered to the tenets of the Declaration of Helsinki and was approved by the Chinese PLA General Hospital.

### Definitions and data collection

For statistical research, the glaucoma patients with MICOF were divided into two groups: preexisting glaucoma and *de novo* glaucoma. The diagnosis of preexisting glaucoma was based on IOP and a history of glaucoma before MICOF. *De novo* glaucoma was diagnosed by an experienced physician according to IOP, VF defect, and CDR after MICOF.

The sex, age, preoperative diagnosis, glaucoma, glaucoma surgery, keratoplasty, times of keratoplasty, preoperative BCVA, postoperative BCVA and final follow-up corrected visual acuity, VF progress, and CDR were statistically analyzed.

Intraocular pressure can be measured by iCare before MICOF. Due to the existence of an optical cylinder, the IOP can only be estimated by experienced physicians pressing the eyeball with their fingers, which was objective. All the patients had poor visual acuity before MICOF and could not be examined by visual field examination. If the postoperative BCVA was higher than 1.0 logarithm of the minimum angle of resolution (logMAR), it was feasible to detect VF. Significant widening of the VF defect or a decrease in the visual field index (VFI) was defined as VF progression. Due to opaque optical media presence in those patients before MICOF, optical coherence tomography (OCT) examination of CDR could not be performed, so only the postoperative OCT data were counted.

According to the data, the preoperative diagnoses included acid burn, alkali burn, thermal burn, explosion injury, autoimmune disease, glaucoma, and keratoconus. Glaucoma surgery is divided into: cyclocryotherapy, endoscopic cyclophotocoagulation, transscleral cyclophotocoagulation, trabeculectomy, and glaucoma drainage device implantation. According to the literature, BCVA was converted into logMAR: 0.1 = 1.0 logMAR, no light perception = 3.0 logMAR, light perception = 2.3 logMAR, hand motion = 2.0 logMAR, and counting fingers = 1.7 logMAR (9).

### Statistical analysis

Data were analyzed using the SPSS software version 22.0. The *t*-test or the Mann–Whitney *U*-test was used to compare the differences in continuous variables between the two groups, and Pearson's chi-square tests or Fisher's exact tests were used to evaluate the differences in the classified variables between the groups. GraphPad Prism version 8 was used to analyze the statistical results.

## Results

### General data of patients

The data showed that 39 eyes (39 patients) had glaucoma, including 33 eyes (84.6%) of males and six eyes (15.4%). of females. The average age of the patients was 48.21 ± 13.33 years old. The follow-up time was 69.38 ± 50.22 months, and the median follow-up time was 62 months.

### Comparison of related data between preexisting glaucoma and *de novo* glaucoma

Table 1 shows the comparison of sex, eye type, age, preoperative diagnosis, keratoplasty, and postoperative complications between the preexisting glaucoma (*n* = 16) and *de novo* glaucoma after MICOF (*n* = 23) (Table 1). The related data of the patients in the two groups were similar. The most common preoperative diagnosis was alkali burns (56.3% of preexisting glaucoma and 43.5% of *de novo* glaucoma). The proportion of the eyes with preexisting glaucoma treated with keratoplasty (56.3%) was higher than that of the eye with *de novo* glaucoma (21.7%, *P* < 0.05).

**Table 1:** Comparison of related data between preexisting glaucoma and *de novo* glaucoma.

	**Preexisting glaucoma**	***De novo*** **glaucoma**	***P*** **-value**
	***n*** **= 16**	***n*** **= 23**	
Male gender	14 (87.5%)	19 (82.6%)	1
Right operated eye	8 (50%)	7 (30.4%)	0.182
Age, years	45.75 ± 12.13	49.91 ± 14.12	0.344
**Preoperative diagnosis**			
Alkali burn	9 (56.3%)	10 (43.5%)	0.523
Acid burn	2 (12.5%)	2 (8.7%)	1
Thermal burn	1 (6.3%)	4 (17.4%)	0.631
Explosion burn	1 (6.3%)	3 (13.0%)	0.638
Autoimmune disease	1 (6.3%)	4 (17.4%)	0.631
Glaucoma	1 (6.3%)	0	0.41
Keratoconus	1 (6.3%)	0	0.41
Keratoplasty	9 (56.3%)	5 (21.7%)	***0.043***
Number of keratoplasty	0.81 ± 0.911	0.45 ± 0.963	0.255
Initial BCVA	2.23 ± 0.13	2.14 ± 0.18	0.139
Postoperative BCVA	0.76 ± 0.61	0.63 ± 0.52	0.412
Best BCVA	0.43 ± 0.58	0.17 ± 0.20	0.107
Final BCVA	1.03 ± 1.00	0.78 ± 0.88	0.374
**Complications**			
Cornea melting	2	10	0.076
Overgrowth of the surface mucosa	3	5	1
Retroprosthetic membrane	1	1	1
Infective endophthalmitis	2	2	1
Aseptic endophthalmitis	1	1	1
Macular edema	0	3	0.255
Retinal detachment	0	1	1

The meaning of the bold + italic values is that the difference is statistically significant.

### Comparison of glaucoma surgery

According to the data, the patients were divided into the preexisting glaucoma group and the *de novo* glaucoma group. Then, the patients with preexisting glaucoma were divided into the preoperative glaucoma operation group (*n* = 8), the intraoperative glaucoma surgery group (*n* = 4), and the medication group (*n* = 4). The patients with *de novo* glaucoma were divided into the glaucoma surgery group (*n* = 7) and the medication group (*n* = 16). Among the preexisting glaucoma group, eight eyes underwent glaucoma surgery before the operation, including cyclocryotherapy (*n* = 3, 37.5%), endoscopic cyclophotocoagulation (*n* = 1, 12.5%), transscleral cyclophotocoagulation (*n* = 1, 12.5%), trabeculectomy (*n* = 1, 12.5%), glaucoma drainage device implantation (*n* = 1, 12.5%), and trabeculectomy combined with glaucoma drainage device implantation (*n* = 1, 12.5%). A total of 4 eyes underwent glaucoma surgery during MICOF surgery, cyclocryotherapy in 2 eyes (50%), endoscopic cyclophotocoagulation in 1 eye (25%), and cyclocryotherapy combined with glaucoma drainage device implantation in 1 eye (25%). A total of four eyes were treated with medication only. Among the patients with *de novo* glaucoma, seven eyes underwent glaucoma surgery, including endoscopic cyclophotocoagulation (*n*=5, 71.4%), transscleral cyclophotocoagulation (*n* = 1, 14.2%), and cyclocryotherapy combined with glaucoma drainage device implantation (*n* = 1, 14.2%). A total of 16 eyes were treated with medication only (Table 2).

**Table 2:** Treatment of the preexisting glaucoma and *de novo* glaucoma.

	**Preexisting glaucoma (*****n*** **= 16)**			***De novo*** **glaucoma (*****n*** **= 23)**	
	**Glaucoma surgery pre- MICOF (*n* = 8)**	**Glaucoma surgery during MICOF (*n* = 4)**	**Medication only (*n* = 4)**	**Glaucoma surgery after MICOF (*n* = 7)**	**Medication only (*n* = 16)**
**Glaucoma surgery**					
Cyclocryotherapy	3 (37.5%)	2 (50%)			
Endoscopic cyclophotocoagulation	1 (12.5%)	1 (25%)		5 (71.4%)	
Transscleral cyclophotocoagulation	1 (12.5%)			1 (14.2%)	
Trabeculectomy	1 (12.5%)				
Glaucoma drainage devices implantation	1 (12.5%)				
Trabeculectomy combined with glaucoma Drainage devices implantation	1 (12.5%)				
Cyclocryotherapy combined with glaucoma drainage devices implantation	1 (25%)		1 (14.2%)	

### Comparison of the glaucoma surgery group and the medication group

The study compared the effects of glaucoma surgery and medication on visual acuity (initial BCVA, postoperative BCVA, best BCVA, and final BCVA), CDR (per year), visual field loss, and complications. There was little difference in the initial BCVA, postoperative BCVA, and best BCVA, but in the *de novo* glaucoma group, the final BCVA in the glaucoma surgery group (1.56 ± 1.07) was worse than that in the medication group (0.44 ± 0.53) (*P* = 0.017). There was no significant difference in CDR or visual field loss. Regarding complications, the incidence of cornea melting in the eyes with *de novo* glaucoma treated with medication only (*n* = 10) was significantly higher than that in the eyes with glaucoma surgery (*n* = 0) (*P* = 0.007). There was no significant difference in the incidence of other complications (Table 3).

**Table 3:** Comparison of the glaucoma surgery group and the medication group.

	**Preexisting glaucoma**			***De novo*** **glaucoma**		
	**Glaucoma surgery pre or during MICOF (*n* = 12)**	**Medication only (*n* = 4)**	***P*** **-value**	**Glaucoma surgery after MICOF (*n* = 7)**	**Medication only (*n* = 16)**	***P*** **-value**
Initial BCVA	2.23 ± 0.14	2.23 ± 0.15	1	2.13 ± 0.24	2.15 ± 0.15	1
Postoperative BCVA	0.85 ± 0.66	0.50 ± 0.43	0.299	0.53 ± 0.45	0.67 ± 0.55	0.437
Best BCVA	0.49 ± 0.64	0.25 ± 0.38	0.389	0.27 ± 0.23	0.13 ± 0.17	0.1
Final BCVA	1.09 ± 0.87	0.83 ± 1.46	0.161	1.56 ± 1.07	0.44 ± 0.53	***0.017***
CDR change/year	0.119 ± 0.103	0.069 ± 0.089	0.18	0.150 ± 0.135	0.096 ± 0.111	0.151
Visual field loss	4 (33.3%)	1 (25%)	1	5 (71.4%)	4 (25%)	0.066
**Complications**					
Cornea melting	1	1	0.45	0	10	***0.007***
Overgrowth of the surface mucosa	3	0	0.529	2	3	0.621
Retroprosthetic membrane	0	1	0.25	0	1	1
Infective endophthalmitis	1	1	0.45	0	2	1
Aseptic endophthalmitis	1	0	1	0	1	1
Macular edema	0	0		1	2	1
Retinal detachment	0	0		1	0	0.304

The meaning of the bold + italic values is that the difference is statistically significant.

### Effect of glaucoma surgery on visual field loss

**Figure 1:**
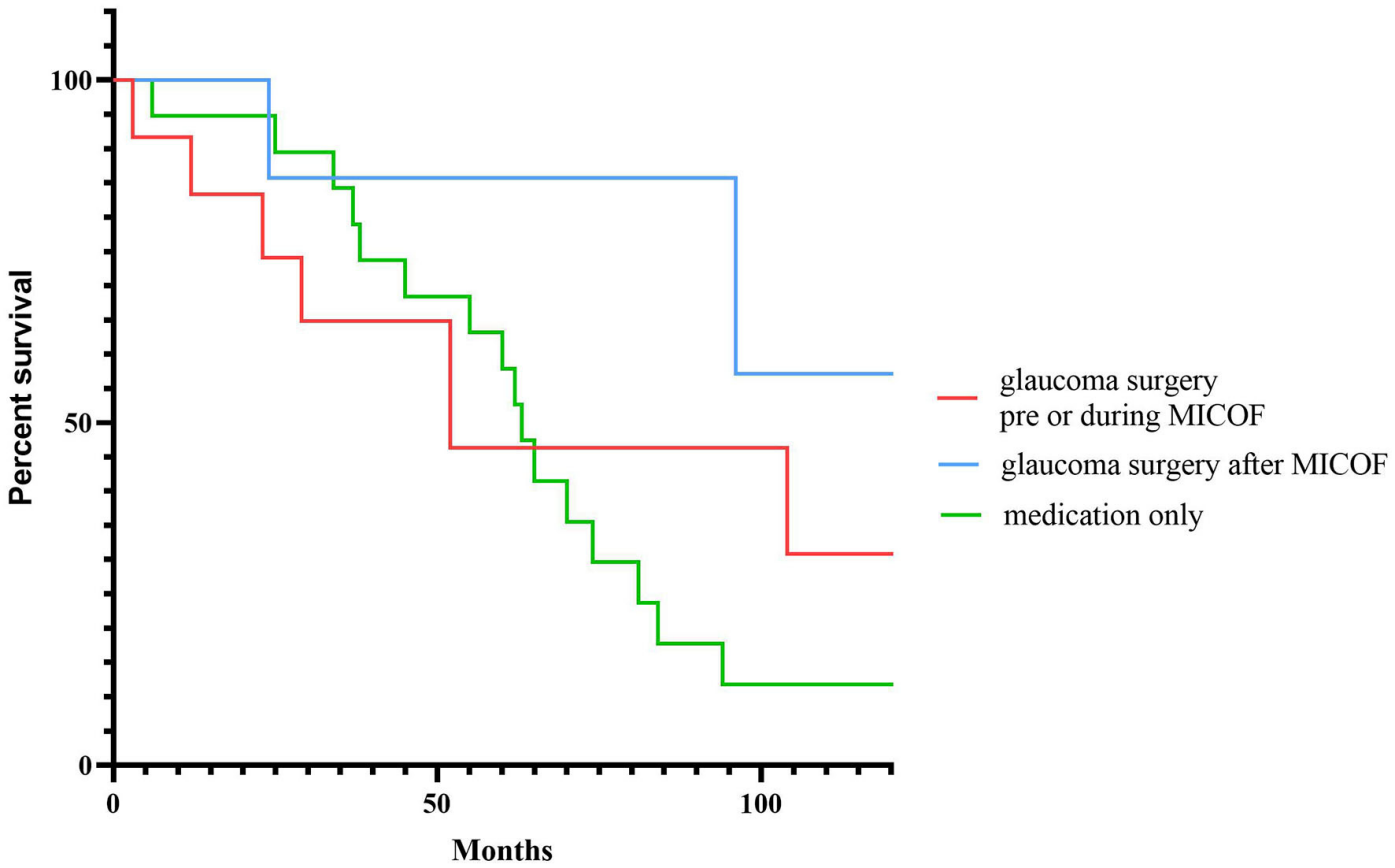
The Kaplan–Meier survival curve for visual field loss to compare the timing of glaucoma surgery and medication.

The Kaplan–Meier survival curve was used to analyze the loss of the VF in the glaucoma surgery pre, during, or after MICOF, and the medication only group (Figure 1).

## Discussion

In recent years, the research and clinical application of keratoprosthesis have become increasingly widespread, which brings hope for the recovery of vision for patients with severe ocular surface diseases. However, glaucoma, like an invisible killer, quietly affects the vision of patients. Studies have shown that before keratoprosthesis implantation, the prevalence of glaucoma is 36–76%, and 8–75% of eyes develop new glaucoma after surgery (10, 11). In the patients with severe ocular surface diseases, such as chemical burns, autoimmune keratopathy, severe dry eyes, and repeated keratoplasty failures, the transparency of the corneal conjunctiva is reduced, which makes the evaluation of glaucoma difficult. After keratoprosthesis in these patients, IOP monitoring becomes very difficult because of the uniqueness of the optical cylinder. Therefore, the prevention and treatment of glaucoma are particularly important to maintain the vision of patients with keratoprosthesis. According to our previous study (12), 17 of 91 patients with MICOF had preexisting glaucoma. Among them, 7 patients developed glaucoma after MICOF, and 16 patients had *de novo* glaucoma after MICOF, which threatened their vision. However, most of the studies are Boston KPro currently. There are few studies on glaucoma in patients with MICOF. Due to the different types and structures of corneal prostheses, the results of other types of KPro cannot be simply applied to patients with MICOF. Therefore, this study used the statistics of patients with MICOF, to explore the timing of glaucoma treatment.

In this study, alkali burns were the most common preoperative diagnosis of glaucoma. Chemical burns, especially alkali burns, are well indications for keratoprosthesis. After keratoprosthesis implantation, patients with alkali burns can obtain better vision in a short time, but the maintenance of vision will be a challenge. Because of its strong penetration ability, alkaline substances can not only damage the ocular surface, but also cause the destruction of intraocular structure and inflammation, which increase the incidence of glaucoma, even if the retina and optic nerve look normal temporarily. However, the retina ganglion cell layer may have been destroyed (13, 14), making it extremely sensitive to IOP, and the loss of vision can be caused without excessive IOP (15). In addition, this study found that the proportion of patients with preexisting glaucoma with previous keratoplasty was higher than that in the patients with *de novo* glaucoma after MICOF, and the difference was statistically significant. Abnormalities in the intraocular structure may be aggravated during keratoplasty (16), and the use of dexamethasone after keratoplasty is also a risk factor for glaucoma (17). Therefore, attention should be given to the evaluation of glaucoma in patients with alkali burns and previous keratoplasty.

To accurately evaluate the treatment and timing of glaucoma, the patients with preexisting glaucoma were divided into three groups: the glaucoma surgery pre-MICOF group, the glaucoma surgery during MICOF group, and the medication only group. The patients with *de novo* glaucoma were divided into the glaucoma surgery after MICOF group and the medication only group. In this study, the patients with preexisting glaucoma were not treated with glaucoma surgery after MICOF. Among the patients with preexisting glaucoma, there was no significant difference in the postoperative BCVA, best BCVA, or final BCVA between the operation group and the medication group, which may indicate that there was previous nerve loss in the patients with glaucoma. As long as IOP can be maintained, surgery has no effect on glaucoma. In the patients with *de novo* glaucoma, the final BCVA in the surgery group was worse than that in the medication group, which may be because the degree of glaucoma in the patients requiring surgical treatment was already more severe than that in the patients treated with drugs. In the study of Boston KPro by Dominique (18), it was found that the eyes with glaucoma surgery after KPro progressed faster than the eyes with glaucoma surgery pre-KPro or medication in the patients with preexisting glaucoma, and the complications did not increase. Therefore, it is recommended to combine glaucoma drainage device implantation during KPro. In this study, regardless of preexisting or *de novo* glaucoma, ciliary body destruction was often used because the eye surface state of patients with MICOF is generally very poor, and the conjunctival condition is not good. Shunt surgery may not have a good effect (19).

Because of the difficulty of IOP measurement in patients with keratoprosthesis, it can only be measured artificially, and there is certain subjectivity. When the refractive stroma is transparent, glaucoma progression can be evaluated by optic disk OCT or visual field progression monitoring. In this study, there was no significant difference in the change in CDR or the progression of the visual field between the preexisting glaucoma group and the *de novo* glaucoma group, the glaucoma surgery group and the medication group. This is consistent with the study of Boston KPro by Dominique (18). Silva (20) found that the narrowing of the temporal chamber angle has diagnostic significance for the progression of Boston KPro glaucoma through anterior segment OCT, but this is based on the premise that the chamber angle imaging is clear.

Regarding complications, the incidence of cornea melting in the medication treatment group was significantly higher than that in the glaucoma surgery group. In terms of the process of operation, the aim for the implantation of MICOF is to make a lamellar cornea pocket to place the titanium frame, and a PMMA optical cylinder was screwed into the frame, the donor cornea which is thin compared to the cornea covered by Boston KPro (21). When the IOP is high, the autologous tissue nutrition is poor, and the pressure of the optical cylinder and frame on the outer corneal tissue increases, which increases the incidence of cornea melting. In addition, persistent inflammation of the ocular surface can also lead to cornea melting (22). Although the follow-up visual acuity in the glaucoma surgery group was worse than that in the medication group, it was observed, by generating the Kaplan–Meier survival curve, that the visual field of early drug treatment and glaucoma surgery after MICOF was slow, according to whether the VF progressed. However, in the long run, there was persistent visual field loss in the medication only group, and the drugs that are ocularly applied may not be absorbed well due to ocular surface scarring.

## Conclusion

According to our study, patients with MICOF who have undergone keratoplasty are more likely to develop glaucoma, and the focus should be on the prevention and treatment. *De novo* glaucoma can be treated by surgery according to the ocular surface condition to reduce the incidence of complications. However, the number of patients included in this study was small, and more clinical data need to be collected for research.

## Data availability statement

The original contributions presented in the study are included in the article/supplementary material, further inquiries can be directed to the corresponding author/s.

## Ethics statement

The study adhered to the tenets of the Declaration of Helsinki and was approved by the Chinese PLA General Hospital, informed consent was obtained from all subjects and/or their legal guardian(s). The patients/participants provided their written informed consent to participate in this study.

## Author contributions

ZL: collected data, analyzed data, and wrote articles. QW: critical correction of the manuscript. S-FZ: collected data. Y-FH and L-QW: critical revision and approval of the article. All authors contributed to the article and approved the submitted version.

## Funding

This study has been funded by the Fund Project of China Joint Logistic Support Force (LB20201A010024).

## Conflict of interest

The authors declare that the research was conducted in the absence of any commercial or financial relationships that could be construed as a potential conflict of interest.

## Publisher's note

All claims expressed in this article are solely those of the authors and do not necessarily represent those of their affiliated organizations, or those of the publisher, the editors and the reviewers. Any product that may be evaluated in this article, or claim that may be made by its manufacturer, is not guaranteed or endorsed by the publisher.

## References

[1] Shimazaki J Shimmura S Tsubota K. Donor source affects the outcome of ocular surface reconstruction in chemical or thermal burns of the cornea. Ophthalmology. (2004) 111:38–44. 10.1016/j.ophtha.2003.02.003 14711712

[2] Meduri A Valastro A Inferrera L Oliverio GW Ninotta I Camellin U. Sutureless amniotic membrane transplantation in inflammatory corneal perforations. Appl Sci. (2022) 12:3924–33. 10.3390/app12083924

[3] Ilhan-Sarac O Akpek EK. Current concepts and techniques in keratoprosthesis. Curr Opin Ophthalmol. (2005) 16:246–50. 10.1097/01.icu.0000172829.33770.d3 16000898

[4] de Oliveira LA Magalhães FP Hirai FE de Sousa LB. Experience with Boston keratoprosthesis type 1 in the developing world. Can J Ophthalmol. (2014) 49:351–7. 10.1016/j.jcjo.2014.05.003 25103652

[5] Hille K Grabner G Liu C Colliardo P Falcinelli G Taloni M. Standards for modified osteoodontokeratoprosthesis (OOKP) surgery according to Strampelli and Falcinelli: the Rome-Vienna Protocol. Cornea. (2005) 24:895–908. 10.1097/01.ico.0000157401.81408.62 16227830

[6] Schrage N Hille K Cursiefen C. [Current treatment options with artificial corneas: Boston Kpro, Osteo-odontokeratoprosthesis, Miro Cornea(R) and KeraKlear(R)]. Ophthalmologe. (2014) 111:1010–8. 10.1007/s00347-013-3009-5 25388085

[7] Geoffrion D Harissi-Dagher M. Glaucoma risk factors and outcomes following boston keratoprosthesis type 1 surgery. Am J Ophthalmol. (2021) 226:56–67. 10.1016/j.ajo.2021.01.006 33493469

[8] Nguyen P Chopra V. Glaucoma management in Boston keratoprosthesis type I recipients. Curr Opin Ophthalmol. (2014) 25:134–40. 10.1097/ICU.0000000000000035 24469078

[9] Schulze-Bonsel K Feltgen N Burau H Hansen L Bach M. Visual acuities “hand motion” and “counting fingers” can be quantified with the freiburg visual acuity test. Invest Ophthalmol Vis Sci. (2006) 47:1236–40. 10.1167/iovs.05-0981 16505064

[10] Netland PA Terada H Dohlman CH. Glaucoma associated with keratoprosthesis. Ophthalmology. (1998) 105:751–7. 10.1016/S0161-6420(98)94034-9 9544652

[11] Crnej A Paschalis EI Salvador-Culla B Tauber A Drnovsek-Olup B Shen LQ. Glaucoma progression and role of glaucoma surgery in patients with Boston keratoprosthesis. Cornea. (2014) 33:349–54. 10.1097/ICO.0000000000000067 24531120

[12] Wang L He X Wang Q Wu T Liu A Huang Y. Long-term outcomes of the MICOF keratoprosthesis surgery. Ocul Surf. (2021) 21:178–85. 10.1016/j.jtos.2021.06.005 34118425

[13] Choi SH Kim MK Oh JY. Glaucoma after ocular chemical burns: Incidence, risk factors, and outcome. Sci Rep. (2020) 10:4763. 10.1038/s41598-020-61822-5 32179804 PMC7076008

[14] Paschalis EI Zhou C Lei F Scott N Kapoulea V Robert MC. Mechanisms of retinal damage after ocular alkali burns. Am J Pathol. (2017) 187:1327–42. 10.1016/j.ajpath.2017.02.005 28412300 PMC5455067

[15] Cade F Grosskreutz CL Tauber A Dohlman CH. Glaucoma in eyes with severe chemical burn, before and after keratoprosthesis. Cornea. (2011) 30:1322–7. 10.1097/ICO.0b013e31821eead6 22001817

[16] Ahmad S Mathews PM Lindsley K Alkharashi M Hwang FS Ng SM. Boston type 1 keratoprosthesis versus repeat donor keratoplasty for corneal graft failure: a systematic review and meta-analysis. Ophthalmology. (2016) 123:165–77. 10.1016/j.ophtha.2015.09.028 26545318

[17] Wu S Xu J. Incidence and risk factors for post-penetrating keratoplasty glaucoma: a systematic review and meta-analysis. PLoS ONE. (2017) 12:e0176261. 10.1371/journal.pone.0176261 28430806 PMC5400257

[18] Geoffrion D Hassanaly SI Marchand M Daoud R Agoumi Y Harissi-Dagher M. Assessment of the role and timing of glaucoma surgery in boston keratoprosthesis type 1 patients. Am J Ophthalmol. (2022) 235:249–57. 10.1016/j.ajo.2021.09.005 34543660

[19] Wang L Huang Y Du G Dong Y Guo H Wang D. Long-term outcomes and complications of Moscow Eye Microsurgery Complex in Russia (MICOF) keratoprosthesis following ocular surface burns: clinical experience in China. Br J Ophthalmol. (2015) 99:1669–74. 10.1136/bjophthalmol-2014-306115 26034080

[20] Silva RN Taniguchi EV Cruzat A Paschalis EI Pasquale LR Colby KA. Angle anatomy and glaucoma in patients with boston keratoprosthesis. Cornea. (2020) 39:713–9. 10.1097/ICO.0000000000002216 31764284

[21] Lee WB Shtein RM Kaufman SC Deng SX Rosenblatt MI. Boston keratoprosthesis: outcomes and complications: a Report by the American Academy of Ophthalmology. Ophthalmology. (2015) 122:1504–11. 10.1016/j.ophtha.2015.03.025 25934510

[22] Crnej A Omoto M Dohlman TH Dohlman CH Dana R. Corneal inflammation after miniature keratoprosthesis implantation. Invest Ophthalmol Vis Sci. (2014) 56:185–9. 10.1167/iovs.14-15884 25515579 PMC4290558

